# High Lithium Storage Performance of Co Ion-Doped Li_4_Ti_5_O_12_ Induced by Fast Charge Transport

**DOI:** 10.3389/fchem.2022.919552

**Published:** 2022-06-28

**Authors:** M. Wang, Y. Chen, C. X. Yang, Y. H. Zeng, P. F. Fang, W. Wang, X. L. Wang

**Affiliations:** ^1^ School of Materials Science and Engineering, Liaoning Technical University, Fuxin, China; ^2^ Key Laboratory of Mineral High Value Conversion and Energy Storage Materials of Liaoning Province, Fuxin, China

**Keywords:** metallic ion doping, Li4Ti5O12, charge transport, lithium storage performance, the microstructure

## Abstract

In this study, Co_3_O_4_-doped Li_4_Ti_5_O_12_ (LTO) composite was designed and synthesized by the hydrothermal reduction method and metal doping modification method. The microstructure and electrochemical performance of the Co_3_O_4_-doped Li_4_Ti_5_O_12_ composite were characterized by XRD, SEM, TEM, electrochemical impedance spectroscopy, and galvanostatic tests. The results showed that Li_4_Ti_5_O_12_ particles attached to lamellar Co_3_O_4_ constituted a heterostructure and Co ion doped into Li_4_Ti_5_O_12_ lattice. This Co ion-doped microstructure improved the charge transportability of Li_4_Ti_5_O_12_ and inhibited the gas evolution behavior of Li_4_Ti_5_O_12_, which enhanced the lithium storage performance. After 20 cycles, the discharge specific capacity reached stability, and the capacity retention maintained 99% after 1,000 cycles at 0.1 A/g (compared to the capacity at the 20th cycle). It had an excellent rate performance and long cycle stability, in which the capacity reached 174.6 mA h/g, 2.2 times higher than that of Li_4_Ti_5_O_12_ at 5 A/g.

## Introduction

Lithium-ion batteries have the advantages of high energy density, high charge transport rate, long cycle life, high security, and no memory effect. Therefore, it has been widely used in the field of consumer electronics and electric vehicles (Chen et al., 2013; Liu et al., 2013; Yang et al., 2015a; Yang et al., 2015b; Liu et al., 2015; Li Z et al., 2016; Li H. Z et al., 2016; Li S et al., 2016; Qu et al., 2018; Lu et al., 2019). Li_4_Ti_5_O_12_ (LTO) was widely studied as anode material for lithium-ion batteries due to its good electrochemical performance (Zhang et al., 2013; Sun et al., 2014; Yan, 2014; You et al., 2018; Wang, 2020; Wang, 2021). However, the low theoretical specific capacity, the low charge transport rate, and the poor electrical conductivity led to serious polarization during rapid charge and discharge, which greatly limited its wide application (Shen et al., 2012; Kim et al., 2013; Zettsu et al., 2014; Tan and Xue, 2018). In recent years, many researchers have carried out several modification studies of pure LTO, including carbon coating, ion doping, and nanocrystallization. (Tang et al., 2009; Cheng et al., 2010; Shi et al., 2011; Li et al., 2013; Ma et al., 2013; Wang et al., 2013; Cheng et al., 2014; Li et al., 2014; Liu et al., 2014; Zhang et al., 2021). In this study, layered Co_3_O_4_ and spherical LTO heterostructures with large specific surface area and short ion diffusion length were prepared by the ion doping method. The composite has excellent electrochemical performance using the microstructure characterization and electrochemical performance test.

## Experimental

**FIGURE 1:**
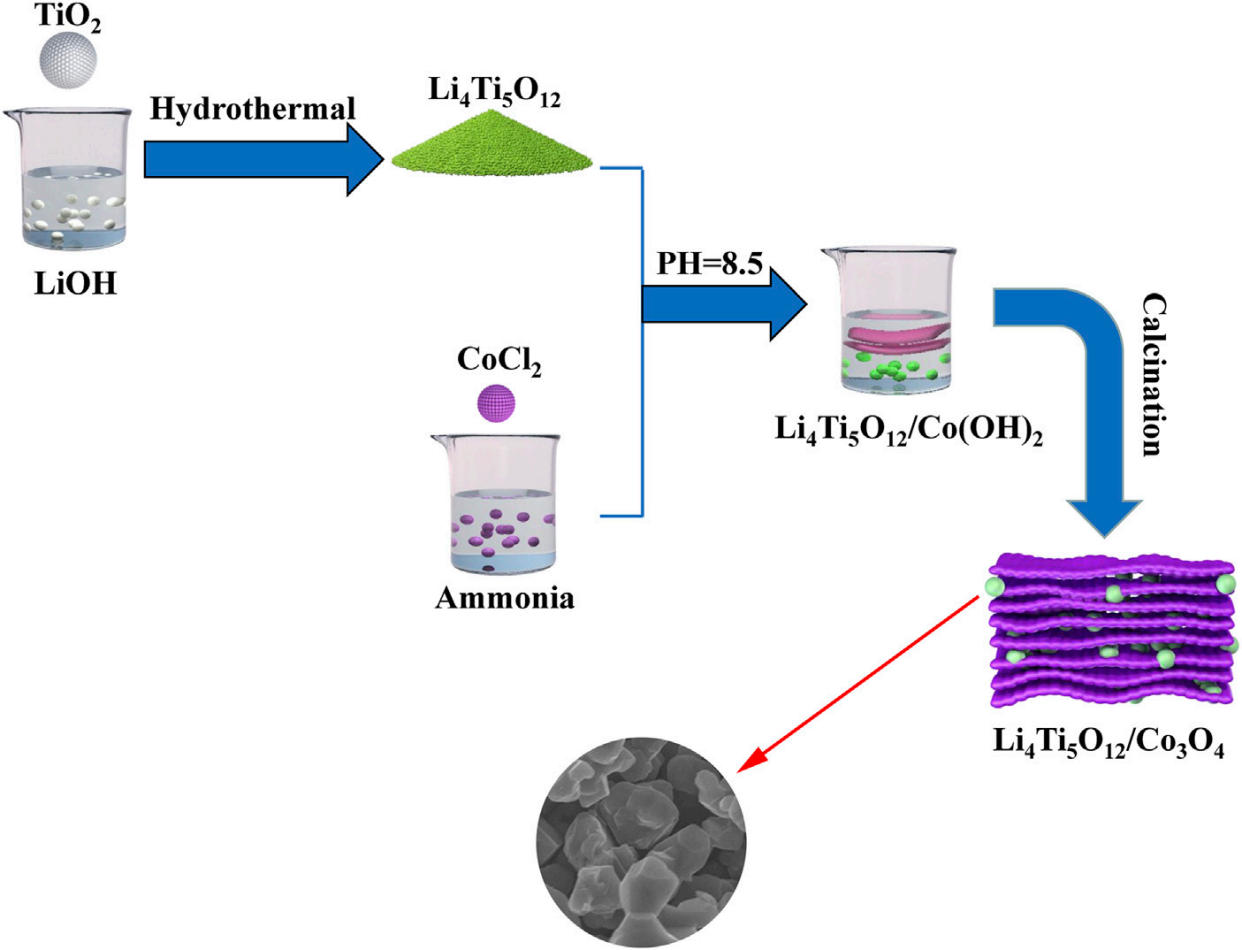
Schematic diagrams of the synthesis of the Li_4_Ti_5_O_12_/Co_3_O_4_ composite.

Firstly, 1.5 mg of CoCl_2_ was dissolved in 30 ml dilute ammonia solution (0.3 mol/L). The pH value of the above solution was adjusted to 8.5 by concentrated ammonia solution and stood for 12 h. The formed precipitate (α-Co(OH)_2_) was filtered and dried. Then, a certain mass of α-Co(OH)_2_, LiOH, and TiO_2_ was mixed and placed in a 100 ml Teflon-lined stainless steel autoclave and heated at 90°C for 12 h. After the temperature was cooled to room temperature, the solution was filtered and dried. The precursors were heated at 800°C for 4 h in a tube furnace. The obtained product was LTO/Co_3_O_4_ powder. Finally, CR2025-type coin cells were assembled in a high-purity Ar-filled ZKX glovebox. The schematic diagram of the synthesis of the LTO/Co_3_O_4_ composite is shown in Figure 1.

The phase composition of the specimen was characterized by XRD (SHIMADZU XRD-6100). The microstructure and morphology of the specimen were analyzed by SEM (JSM-7500F) and FEI TEM (Tecnai G2T20). The charge and discharge performance, rate performance, cycle performance, and Coulombic efficiency, among others, were tested on the battery performance test system (NEWARE). Electrochemical impedance spectroscopy (EIS) was tested on the CHI660E electrochemical workstation.

## Results and Discussion

**FIGURE 2:**
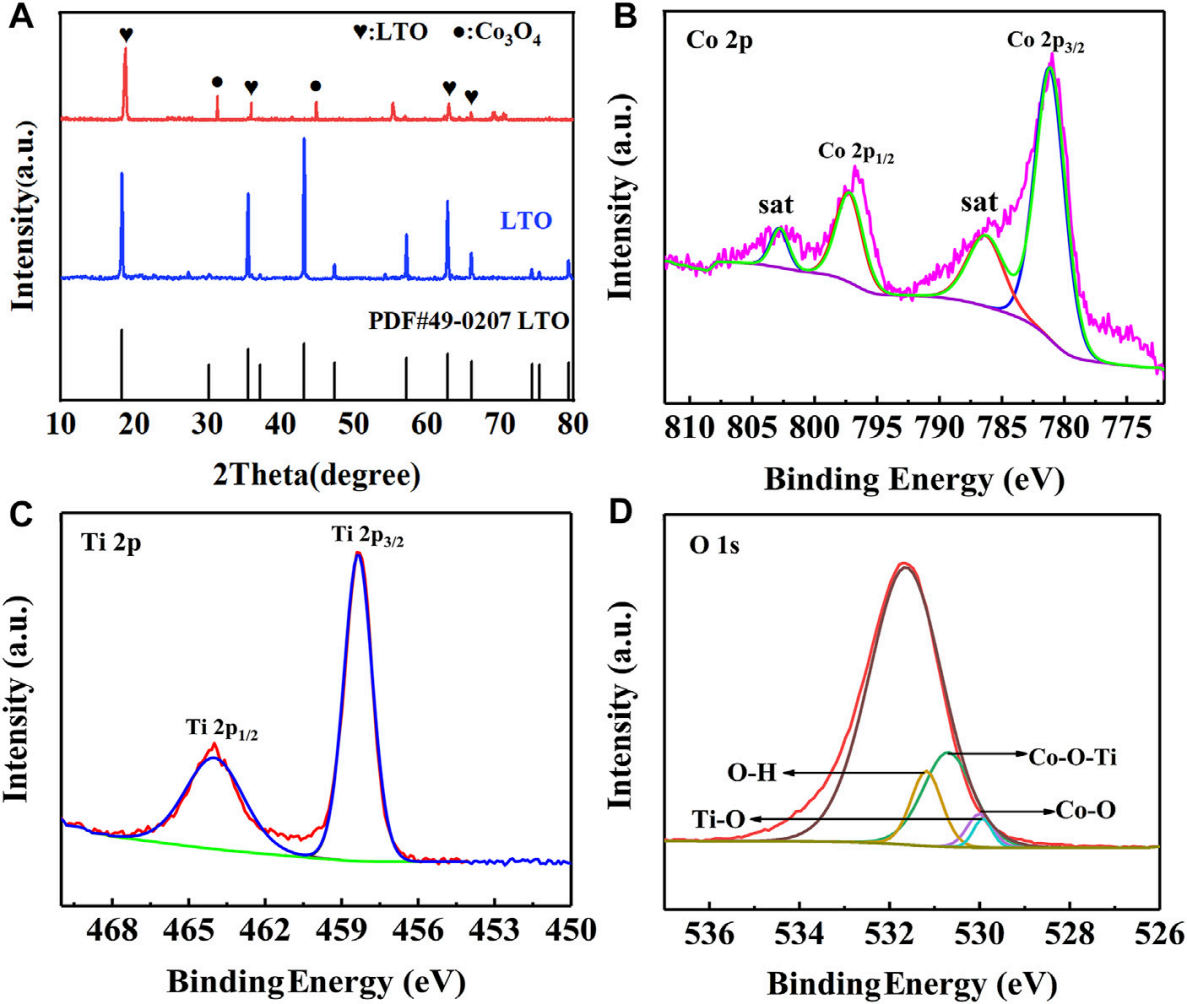
**(A)** XRD patterns of Li_4_Ti_5_O_12_ and Li_4_Ti_5_O_12_/Co_3_O_4_ composite. **(B–D)** XPS patterns of Li_4_Ti_5_O_12_/Co_3_O_4_ composite. **(B)** Co 2p; **(C)** Ti 2p; **(D)** O 1s.

The XRD pattern of LTO and LTO/Co_3_O_4_ composites prepared by the hydrothermal method is shown in Figure 2. It was found that the diffraction peak of the LTO/Co_3_O_4_ composite at 18.3°, 35.6°, 62.8°, and 66.1° corresponded to the crystal planes of (111), (311), (440), and (531), respectively. The characteristic diffraction peak of Co_3_O_4_ at 31.3° and 44.8° corresponded to the crystal planes of (220) and (440), respectively. In addition, it was observed that the diffraction peak of the LTO/Co_3_O_4_ composite shifted significantly to the right. For the (111) crystal plane of LTO, when Co ions were doped into the LTO lattice, the diffraction peak of the composite shifted to the right at approximately 0.5°; the reason for the radius of the Co atom (1.26 Å) was less than that of the Ti atom (1.45 Å), indicating that LTO and Co_3_O_4_ have a good combination.

The surface chemical composition and interfacial bonding state of the LTO/Co_3_O_4_ composite were analyzed by XPS, as shown in Figure 2. The high-resolution spectra of Co 2p, Ti 2p, and O 1s are shown in Figures 2B–D, respectively. It can be seen from Figure 2B that the two signal peaks located at 795.7 and 779.8 eV corresponded to the Co 2p_3/2_ and Co 2p_1/2_ of Co 2p, respectively, and each diffraction peak was accompanied by a satellite peak (Yang et al., 2022). The two characteristic peaks of Ti 2p at 464.3 and 458.6 eV were the spin-orbital peaks of Ti 2p_1/2_ and Ti 2p_3/2_, respectively (see Figure 2C), which was consistent with Zhou’s results (Zhou et al., 2006). In addition, Figure 2D shows the Ti-O, Co-O, O-H, and Co-O-Ti bonds, four diffraction peaks, corresponding to 529.9, 530.0, 532.1, and 530.7 eV, respectively. The formation of the Co-O-Ti bond was successfully induced by hydrothermal synthesis of the LTO/Co_3_O_4_ composite, which was consistent with the reports in the literature (Xu et al., 2020). There is a synergistic effect between the surface of LTO and Co_3_O_4_, which can effectively improve the electrochemical performance of the composite. At the same time, the unsaturated O atoms in the composite combined with H atoms in water to form an O-H bond. It can also be seen from Figure 2D that the peak intensity of the Ti-O bond was significantly lower than that of the Co-O-Ti bond; the reason for the formation of the Co-O-Ti bond weakened the Ti-O bond, indicating that Co ions were successfully doped into LTO lattice.

**FIGURE 3:**
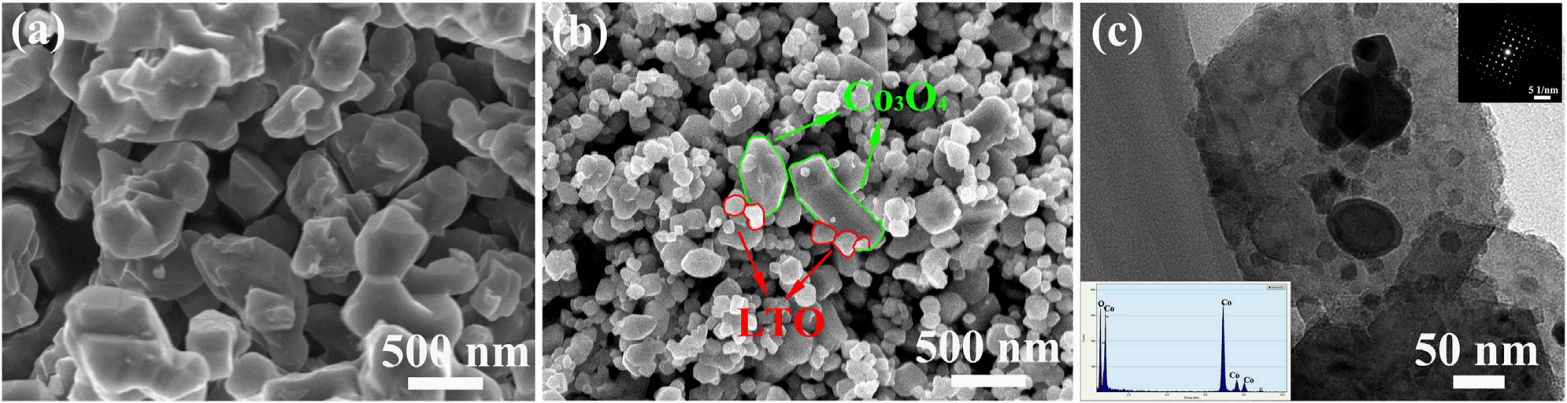
**(A)** SEM image of Li_4_Ti_5_O_12_ particles; **(B)** SEM image of Li_4_Ti_5_O_12_/Co_3_O_4_ composites; **(C)** TEM morphology of Li_4_Ti_5_O_12_/Co_3_O_4_ composites (inset: EDS of the Co_3_O_4_ sheet and SAED pattern of Li_4_Ti_5_O_12_ particle).

Figure 3 shows the SEM morphology and the TEM morphology of LTO and LTO/Co_3_O_4_ composite. Figure 3A shows that the diameters of pure LTO nanoparticles were approximately 200 nm. It can be seen from Figure 3B that LTO spherical nanoparticles were uniformly attached to the surface and interlayer of Co_3_O_4_, in which the particle size of LTO was approximately 50 ± 20 nm and the lamellar diameter of Co_3_O_4_ was approximately 150 ± 50 nm. The addition of Co_3_O_4_ effectively inhibited the growth of LTO nanoparticles. The grain refinement would improve the specific surface area of the composite (Li et al., 2018). Figure 3C shows that the LTO nanoparticles were uniformly dispersed on the layered surface of Co_3_O_4_, indicating that LTO and Co_3_O_4_ combined well. In addition, EDS analysis showed that the composite contained Co and O elements, indicating the existence of Co_3_O_4_ in the composite (see the inset of Figure 3C).

**FIGURE 4:**
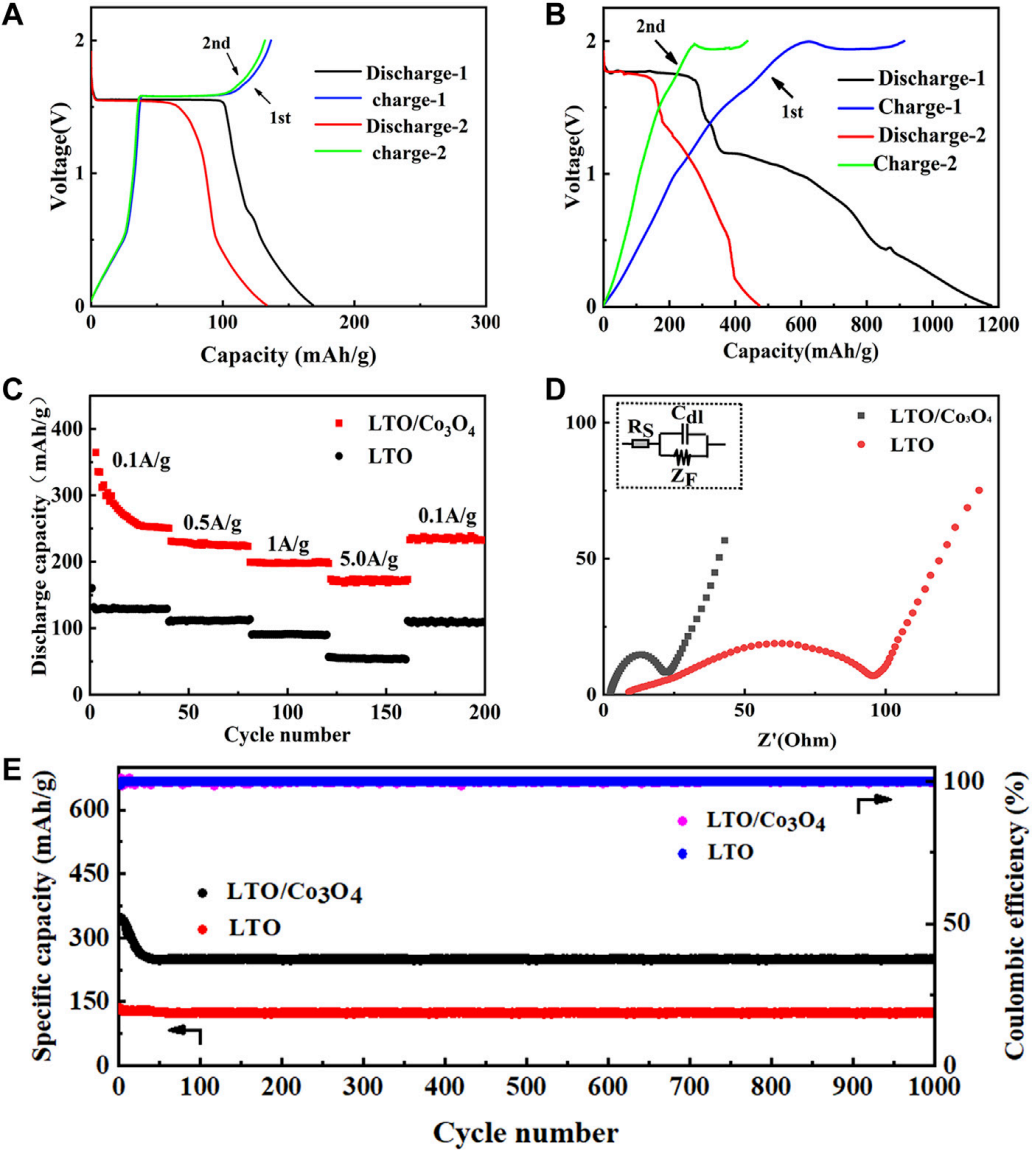
**(A)** The first and second charge/discharge curves of Li_4_Ti_5_O_12_. **(B)** The first and second charge/discharge curves of Li_4_Ti_5_O_12_/Co_3_O_4_. **(C)** The rate performance comparison of Li_4_Ti_5_O_12_ and Li_4_Ti_5_O_12_/Co_3_O_4_. **(D)** The EIS (AC impedance) diagram of Li_4_Ti_5_O_12_ and Li_4_Ti_5_O_12_/Co_3_O_4_. **(E)** Cycle performance curves comparison of Li_4_Ti_5_O_12_ and Li_4_Ti_5_O_12_/Co_3_O_4_ at 0.1A/g.

The first and second charge/discharge curves of LTO and LTO/Co_3_O_4_ composite at 0.1 A/g are shown in Figures 4A,B, respectively. It can be seen that the first discharge specific capacity of LTO and LTO/Co_3_O_4_ composite was 175 and 1,178.0 mA h/g, and the first Coulomb efficiency was 76.3% and 77.6%, respectively. The addition of Co_3_O_4_ improved the ion diffusion rate of the composite, increasing the first discharge specific capacity of the composite. In addition, the second discharge specific capacity of LTO and LTO/Co_3_O_4_ composite was 133.2 and 473 mA h/g, respectively. The first and second discharge specific capacity of LTO and LTO/Co_3_O_4_ composite was quite different. The reason was that the anode material would form SEI film at the electrode/electrolyte interface after the first cycle, which consumed part of Li^+^, causing irreversible capacity loss. Compared with LTO (1.55 V vs. Li/Li^+^ (Wang et al., 2016)), the discharge voltage platform of the LTO/Co_3_O_4_ composite was 1.75 V (vs. Li/Li^+^). The higher discharge voltage platform was beneficial in inhibiting the growth of lithium dendrites and forming a stable SEI film, which improves the cycle performance of the composite. Figure 4C shows the rate performance of LTO and LTO/Co_3_O_4_ composite for 200 cycles at different current densities. The discharge specific capacity of LTO/Co_3_O_4_ composite was higher than that of LTO at different current densities, indicating better rate performance. The discharge specific capacity of LTO at 20, 60, 100, and 140 cycles corresponded to 128.8, 110.9, 91.1, and 53.6 mA h/g, respectively. After 160 cycles, the discharge specific capacity was stable at 111.1 mA h/g, and the capacity retention rate was 86.3% (compared to the capacity at the 20th cycle). The discharge specific capacity of LTO/Co_3_O_4_ at 20, 60, 100, and 140 cycles corresponded to 274.5, 226.2, 201.1, and 174.6 mA h/g, respectively. After 160 cycles, the discharge specific capacity was stable at 230.6 mA h/g, and the capacity retention rate was 84% (compared to the capacity at the 20th cycle). The EIS (AC impedance) test results of LTO and LTO/Co_3_O_4_ composite are shown in Figure 4D. The curve in Figure 4D was fitted by an analog circuit, where R_S_ is ohmic resistance, C_dl_ is the double capacitance between electrode and electrolyte, and Z_F_ is the series connection between R_CT_ (charge transfer resistance) and Z_W_ (Warburg resistance). The results showed that the internal resistance of LTO and LTO/Co_3_O_4_ composite was 9.0 and 2.5 Ω, and the charge transfer resistance was 95.4 and 19.5 Ω, respectively. Compared with pure LTO, the LTO/Co_3_O_4_ composite has lower resistance because the incorporation of Co_3_O_4_ provided more charge transfer channels, improving the charge transport rate of the LTO/Co_3_O_4_ composite. The long cycle performance of the LTO/Co_3_O_4_ composite at 0.1 A/g for 1,000 cycles is shown in Figure 4E. Figure 4E shows that the discharge specific capacity of the composite decreased significantly in the first 20 cycles due to the continuous formation of SEI, leading to the continuous decomposition of Li^+^. With the increase in the cycle number, the SEI film gradually tended to be stable and the discharge specific capacity loss was smaller. After 1,000 cycles, the discharge specific capacity of the LTO and LTO/Co_3_O_4_ composite was maintained at 124.3 and 248.4 mA h/g, and the capacity retention rate reached 96.5% and 99% (compared to the capacity at the 20th cycle), respectively. The LTO/Co_3_O_4_ composite combined with the advantage of Co_3_O_4_ (the high discharge specific capacity) and LTO (the good cycle stability).

The geometric structure model of LTO, Co_3_O_4_, and LTO/Co_3_O_4_ was optimized based on the density functional theory. As shown in Supplementary Figure S1A, 0 eV was defined as the Fermi level. The bandgap between the conduction band and the valence band was 0.8 eV, indicating that the composite exhibited semi-metallic properties. In addition, the energy value of the LTO/Co_3_O_4_ composite was higher than that of LTO and Co_3_O_4_ at the Fermi level (see Supplementary Figure S1B). The synergistic effect between LTO and Co_3_O_4_ significantly increased the probability of electrons appearing in the LTO/Co_3_O_4_ composite at the Fermi level, which was more conducive to electron transfer, improving the charge transfer rate of the LTO/Co_3_O_4_ composite.

## Conclusion

The Co ion-doped LTO composite was prepared using the hydrothermal method. The combination of LTO and Co_3_O_4_ by the Co-O-Ti bond not only maintained the structural stability of the composite but also improved the electron/ion diffusion rate of the composite. Compared with LTO, LTO/Co_3_O_4_ has a higher first discharge specific capacity, good rate performance, and better cycle stability. The first specific capacity was 1,178 mA h/g at 0.1 A/g. After 1,000 cycles, the discharge specific capacity was 248.4 mA h/g and the capacity retention rate was 99% (compared to the capacity at the 20th cycle). At the same time, the LTO/Co_3_O_4_ composite also has a higher discharge specific capacity at high current density (the discharge specific capacity was 174.6 mA h/g at 5 A/g), which was 2.2 times that of pure LTO.

## Data Availability

The original contributions presented in the study are included in the article/Supplementary Material. Further inquiries can be directed to the corresponding author.

## Supplementary Material

Supplementary Figure S1
**(A)** The energy band diagram of Li_4_Ti_5_O_12_/Co_3_O_4_ and **(B)** the density of states of Li_4_Ti_5_O_12_, Co_3_O_4_, and Li_4_Ti_5_O_12_/Co_3_O_4_.
