# Supplementary figures and images for: High Lithium Storage Performance of Co Ion-Doped Li4Ti5O12 Induced by Fast Charge Transport
Source: Front Chem. 2022 Jun 28;10:919552. doi: 10.3389/fchem.2022.919552 (PMC9274094; doi:10.3389/fchem.2022.919552)

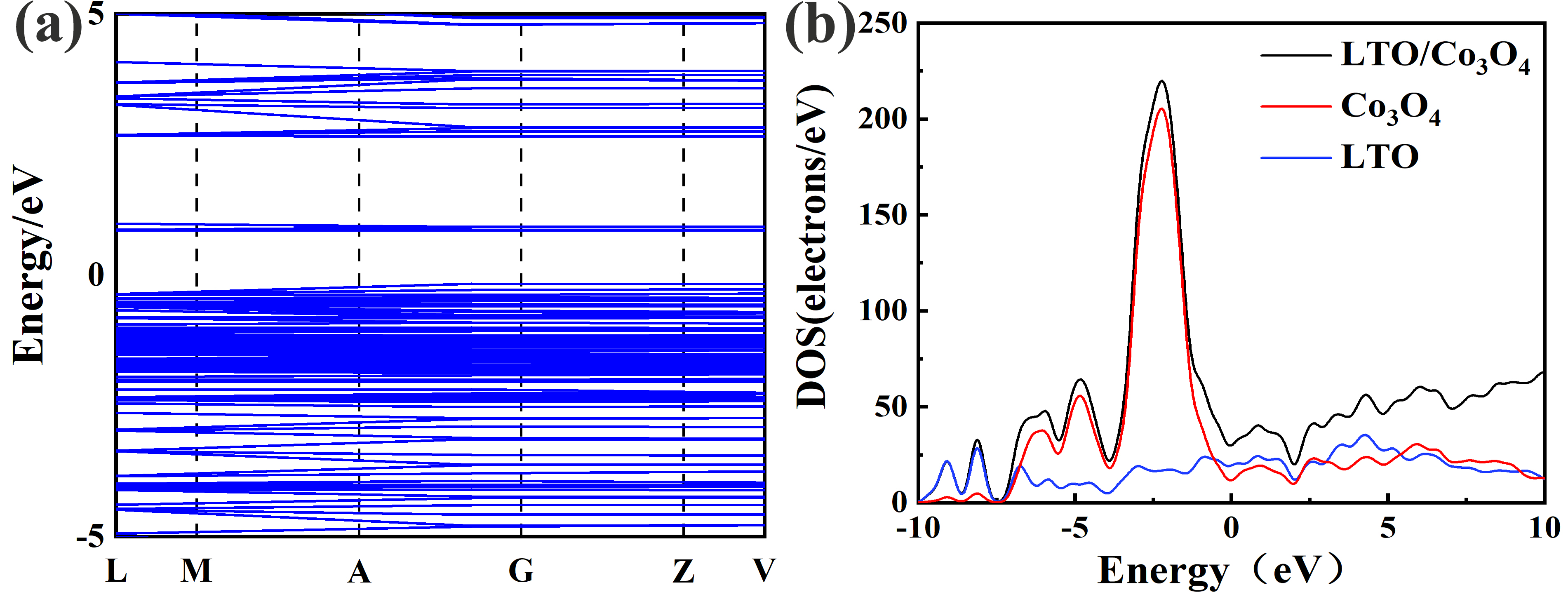

Supplement: Supplementary file 1 [file Image1.TIF]
